# New occurrence of *Meloidogyne graminicola* (Nematoda: Meloidogyninae) from rice fields in Italy: Variability and phylogenetic relationships

**DOI:** 10.1002/ece3.9326

**Published:** 2022-09-20

**Authors:** Elena Fanelli, Francesca Gaffuri, Alberto Troccoli, Stefano Sacchi, Francesca De Luca

**Affiliations:** ^1^ Istituto per la Protezione Sostenibile delle Piante, Bari Consiglio Nazionale delle Ricerche Bari Italy; ^2^ Laboratorio Fitosanitario Regione Lombardia presso Fondazione Minoprio Vertemate con Minoprio Italy

**Keywords:** cytochrome c oxidase subunit I (COI), cytochrome c oxidase subunit II (COII), maximum likelihood, ribosomal DNA, rice, root‐knot nematode

## Abstract

Since the first detection of *Meloidogyne graminicola* in Piedmont, North Italy, in 2016, further inspections for the presence of the rice root‐knot nematode were carried out in rice fields of neighboring regions, in accordance with the Italian NPPO (National Plant Protection Organization) to support the official phytosanitary measures, to enable the early detection of the rice pest, and to prevent its spread within the national territory. In 2018, surveys of rice fields in Lombardy region revealed a new occurrence of *M. graminicola*. In the present study, we confirmed the identification of the rice nematode in Lombardy using the ribosomal ITS region and the mitochondrial COI and COII genes. The sequences and phylogenetic analyses revealed that Lombardy *M. graminicola* population grouped in all trees in the main cluster containing *Meloidogyne* species belonging to *graminis* group, but always in a different subgroup compared to the Piedmont population of *M. graminicola*. These results clearly suggest that the two Italian populations have been recently and independently introduced and confirm that the geographic origin is not the main factor leading to *M. graminicola* population variability.

## Graphical abstract

The manuscript concerns the molecular identification of the rice root‐knot nematode *Meloidogyne graminicola*, included in the EPPO Alert List, occurred in the North of Italy for the second time. The aim of this study is to characterize the population of rice nematode from Lombardy at ecological, evolutionary, and geographic origin level. Understanding the phylogenetic relationships among the different populations and above all between the two Italian populations can also help to address how these populations spread in Italy and the variability observed among them.

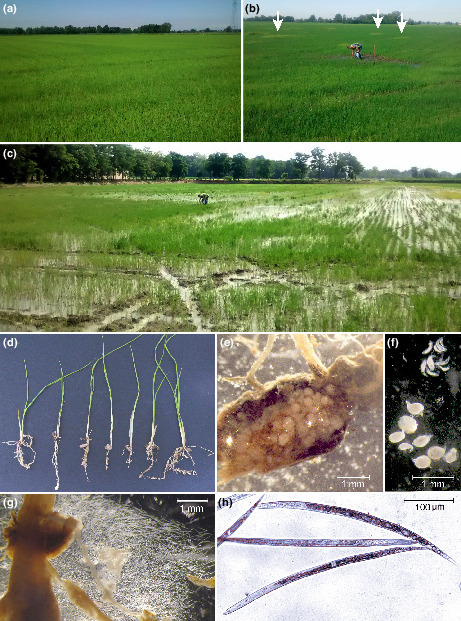

## 1 INTRODUCTION

Root‐knot nematodes are widely distributed causing tremendous economic losses estimated about 80 billion dollars annually (Nicol et al., 2011; Jones et al., 2013). Among *Meloidogyne* species, the root‐knot nematode *Meloidogyne graminicola* Golden and Birchfield (1965) is the most important rice pest worldwide causing yield losses up to 87% (Dutta et al., 2012). *Meloidogyne graminicola* is a nematode species well adapted to attack different rice agrosystems, from upland to lowland, and irrigated to deep‐water fields, along with more than 98 host plants including cereals and grasses (Pokharel et al., 2010). Khan (2015) reported that rice can be attacked by more than 35 plant parasitic nematodes, but *M. graminicola* together with few other *Meloidogyne* spp. is one of the most adapted species to the flooded rice systems, worldwide. Recently, during a survey in Brazil, *M. graminicola* was detected in rice fields along with other *Meloidogyne* spp. belonging to the *graminis* group (Leite et al., 2020; Mattos et al., 2018). It is present in America, Africa, especially in Asia and recently in South Europe (EPPO, 2016, 2018; Fanelli et al., 2017; Mantelin et al., 2017; Rusinque et al., 2021; Sacchi et al., 2021). In Europe, *M. graminicola* was detected for the first time in upland and lowland rice fields in the Piedmont region (Northern Italy) in 2016 and immediately added to the EPPO Alert List. In just one year (2016–2017, EPPO Global Database, 2019), the total infected area increased by fivefold. Italy is the main rice‐growing country in Europe, with 217,195 ha of rice (Ente Nazionale Risi, 2018). The most important rice‐growing area is the section of the Po River Valley straddling the regions of Lombardy and Piedmont with more than 202,000 hectares representing 93% of the Italian rice surface (Ente Nazionale Risi, 2018; Fanelli et al., 2017; Sacchi et al., 2020, 2021).

In Lombardy region, rice growing is highly specialized and is concentrated above all in the area enclosed between Pavia, Milano, and Lodi provinces. Rice fields constitute a typical element of this territory characterized by a large inter‐annual variability related to the meteorological conditions (Zampieri et al., 2019). The Lombardy's rice fields have sandy soils thus draining faster than in Piedmont during summer, the water table is lower, and larger withdrawals from rivers and channels are needed for rice paddy fields irrigation. Therefore, different water management evolution was observed with the increase of spread, in Lombardy (+69%), compared to a lower increase (+28%) in Piedmont, of rice cultivation in dry paddy fields (Torrini et al., 2020; Zampieri et al., 2019). Conditions such as those described (dry paddy fields and sandy soils) can potentially favor the activity of root‐knot nematodes. Therefore, the discovery of *M. graminicola* in Piedmont has causing great concern so as to increase the surveillance by the Lombardy RPPO (Regional Plant Protection Organization) that reported the discovery of the first outbreak in 2018 (EPPO, 2018).

The best phytosanitary measure adopted by the Italian RPPO in Piedmont to control the spread of the *M. graminicola* population was the rice field flooding but, in Lombardy region, this practice is scarcely applicable due to the described soil structure characterized by a low water retention capacity and water shortage (Sacchi et al., 2021; Zampieri et al., 2019). Thus, rapid and accurate identification of *Meloidogyne* spp. associated with rice, specifically *M. graminicola*, as well as prevalence and distribution is important for adopting management strategies in the fields in Northern Italy. In the present study, a population of *M. graminicola* from Lombardy was characterized at molecular level in order to establish the phylogenetic relationships with the Piedmont population and other geographical isolates and to determine the origin of both Italian populations by sequencing the nuclear ITS containing region and the mitochondrial cytochrome oxidase I (COI) and the COII/16S rRNA genes.

## 2 MATERIALS AND METHODS

### 2.1 Nematode isolation

**FIGURE 1:**
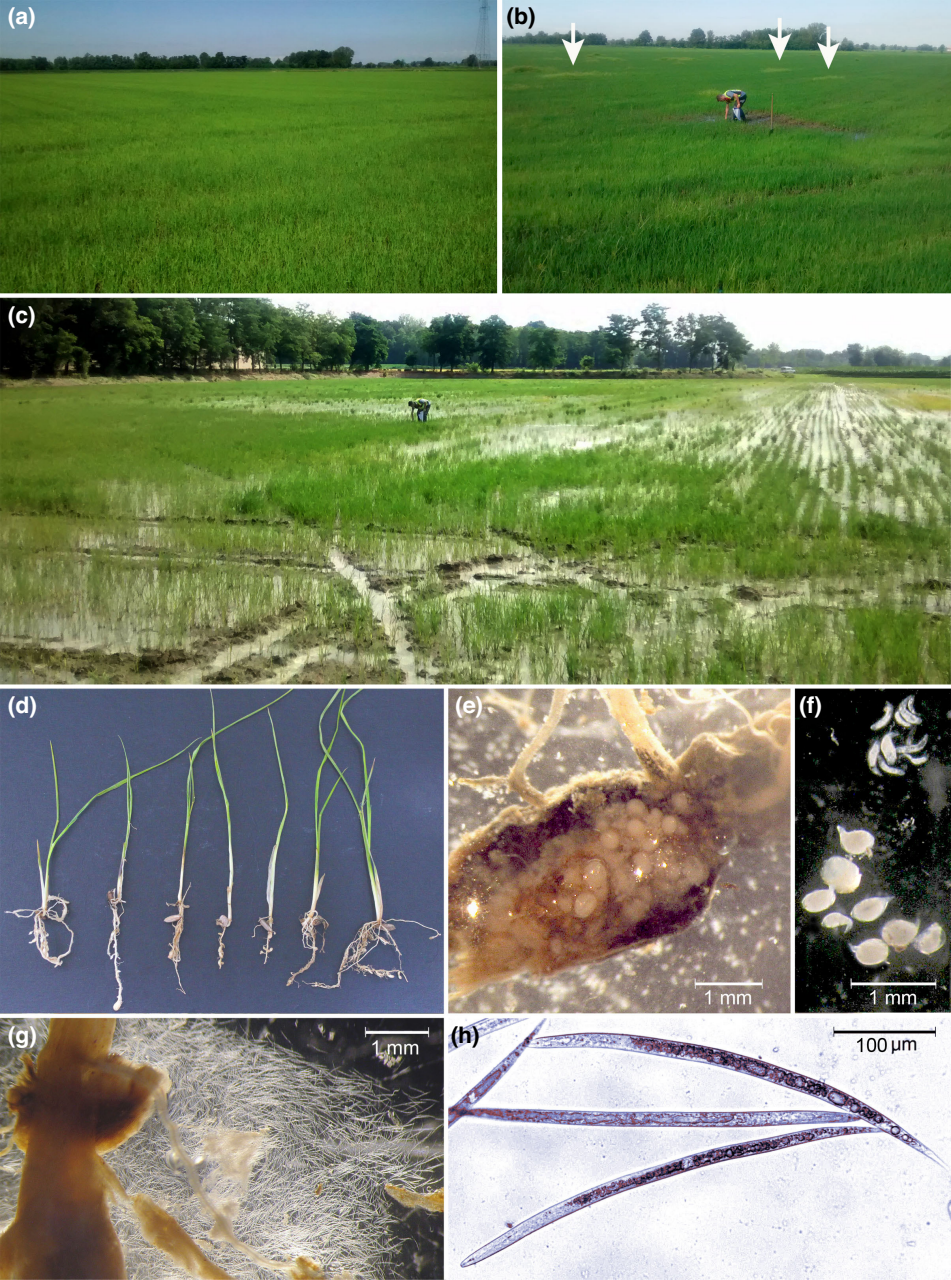
(a) Uninfected rice field; (b) Rice field sowing few patchy areas (arrows); (c) Rice field severely infested by *Meloidogyne graminicola*; (d) Poorly growth rice plantlets showing root tip thickenings caused by massive attacks of *Meloidogyne graminicola*; (e) Open galled rice root showing numerous root‐knot nematode females; (f) Second and third juvenile stages (top), and females (bottom) of *Meloidogyne graminicola* extracted from infected rice roots; (g) Second‐stage juveniles came out from dissected rice roots; (h) Second‐stage juveniles of *Meloydogyne graminicola* from Lombardy.

In 2018, surveys in rice fields showing symptoms of *M. graminicola* attacks (Figure 1) were carried out in several areas of Pavia province (Lombardy). Soil and root samples from the rice‐cultivated area at Cascina Scalina farm in Garlasco (Pavia province) were collected (GPS coordinates: 45.19568654643237, 8.893816095789944) and processed. Second‐stage juveniles, females and males were collected from rice galled roots by direct dissection (EPPO PM 7/119 [1] 2013) under LEICA MZ12 stereomicroscope with Canon PowerShot G3 camera. Morphological identification was made on freshly mounted specimens (second‐stage juveniles or J2 and adults) and female perineal patterns, to a compound microscope ZEISS AXIOSKOP 40 with TrueChrome HD II digital camera and TCapture software (TUCSEN®) for image capture and analysis. Ethanol‐preserved second‐stage juveniles were sent to IPSP‐Bari Institute for molecular identification.

### 2.2 DNA extraction, PCR amplification, and sequencing

Total DNA was extracted from individual nematodes sent to IPSP‐Nematology Lab and directly amplified as described by De Luca et al. (2004). The portion of the ITS1‐5.8S‐ITS2 regions was amplified using the forward primer 18S (5‐TGATTACGTCCCTGCCTTT‐3) and the reverse primer 26S (5′‐TTTCACTCGCCGTTACTAAGG‐3′) (Vrain et al., 1992), the portion of the *COI* gene was amplified as described by Derycke et al. (2005) using forward primer JB3 (5′‐TTTTTTGGGCATCCTGAGGTTTAT‐3′) and reverse primer XiphR1 (5′‐ACAACCAGTTAATCCTCCTACC‐3′) (Lazarova et al., 2006), and the region of the mitochondrial genome between the cytochrome oxidase subunit II (COII) and the 16S rRNA genes was amplified using the forward primer C2F3 (5′‐GGTCAATGTTCAGAAATTTGTGG‐3′) and the reverse primer 1108 (5′‐TACCTTTGACCAATCACGCT‐3′) (Powers & Harris, 1993).

PCR cycling conditions used for amplification of the partial ITS were: an initial denaturation at 94 °C for 3 min, followed by 35 cycles of denaturation at 94 °C for 50 s, annealing at 55 °C for 50 s and extension at 72 °C for 1 min, and a final step at 72 °C for 7 min. For the COI, these conditions were: an initial denaturation at 94 °C for 3 min, followed by 45 cycles of denaturation at 94 °C for 30 s, annealing at 48 °C for 30 s and extension at 72 °C for 30 s, and a final step at 72 °C for 7 min. For the COII, PCR conditions were: an initial denaturation at 94 °C for 3 min, followed by 45 cycles of denaturation at 94 °C for 30 s, annealing at 48 °C for 30 s and extension at 60 °C for 30 s, and a final step at 72 °C for 7 min. 10 J2s of Piedmont *M. graminicola* from upland rice fields were also processed and amplified using ITS and COII primers to confirm the species occurrence in 2018. PCR products of two specimens for each molecular marker were purified after amplification using NucleoSpin (Macherey‐Nagel), quantified using a Nanodrop spectrophotometer (Nanodrop Technologies) and used for cloning in pGEM‐T easy vector (Promega). Eight COI, 5 COII, and 1 ITS clones of *M. graminicola* from Lombardy were sent at MWG‐Eurofins genomics in Germany for sequencing in both directions. Three ITS and three COII clones from Piedmont upland and lowland rice fields were also sent for sequencing to MWG‐Eurofins genomics in the present study.

### 2.3 Phylogenetic analysis

A Basic Local Alignment Search Tool (BLAST) search at National Center for Biotechnology Information (NCBI) was performed in order to confirm their nematode origins (Altschul et al., 1997). The newly obtained sequences for ITS containing region and the partial mitochondrial COI and COII were aligned using MAFFT V.7.450 (Katoh et al., 2019). BioEdit program V. 7.2.5 (Hall, 1999) was used for sequence alignments visualization and edited in order to improve the multialignment. Outgroup taxa, *M. incognita*, *M. hapla*, *M. javanica*, and *M. arenaria*, for each dataset were chosen according to the results of previously published data (Fanelli et al., 2017; Mattos et al., 2018; Soares et al., 2020). Phylogenetic trees, obtained for ITS, COI, and COII dataset, were performed with Maximum Likelihood (ML) method using MEGA version 7 software (Kumar et al., 2016). The phylograms were bootstrapped 1000 times to assess the degree of support for the phylogenetic branching indicated by the optimal tree for each method. The newly obtained sequences were submitted to GenBank with the following accession numbers: for ITS OM809713‐OM809716; for COI OM810293‐OM810300; for COII OP024528‐OP024535.

### 2.4 Estimations of evolutionary divergence between sequences

The pairwise distances within the COI and COII sequences of *M. graminicola* belonging to the *graminis*‐group were done in MEGA7 software package (Kumar et al., 2016). All positions with gaps and missing data were excluded. The COI and COII analyses involved 28 and 25 nucleotide sequences, respectively.

## 3 RESULTS

The PCR products of ITS, mitochondrial COI and COII‐16rRNA yielded fragments of 876 bp, 367 bp, 732 bp, respectively, by sequencing. BLAST search revealed that *M. graminicola* sequences from Lombardy were identical to *M. graminicola* present at NCBI for all genes. ITS region of *M. graminicola* Lombardy population showed 97–99% similarity (1–24 bp different; 0–13 gaps) with all ITS of *M. graminicola* present in the database, in particular the highest identity (99%) was with those from China and India. It is interesting to note that ITS sequences of Lombardy population showed 98% similarity (22–24 bp different; 13 gaps) with the ITS sequences of *M. graminicola* from Piedmont (North Italy). Furthermore, it is noteworthy that *M. graminicola* population from Lombardy and Piedmont showed 98% similarity with the ITS sequences of *M. oryzae* populations from Suriname and French Guiana (LS974439, LS974440, and LS974441; 18 nucleotides and 13 gaps) released in Genbank (Besnard et al., 2019). More recently, other two ITS sequences of *M. oryzae* from Brazil (KY962653, KY962654; Mattos et al., 2018) were released in Genbank and pairwise comparisons showed that both Piedmont and Lombardy *M. graminicola* populations have a 92 and 93% similarity, respectively.

**FIGURE 2:**
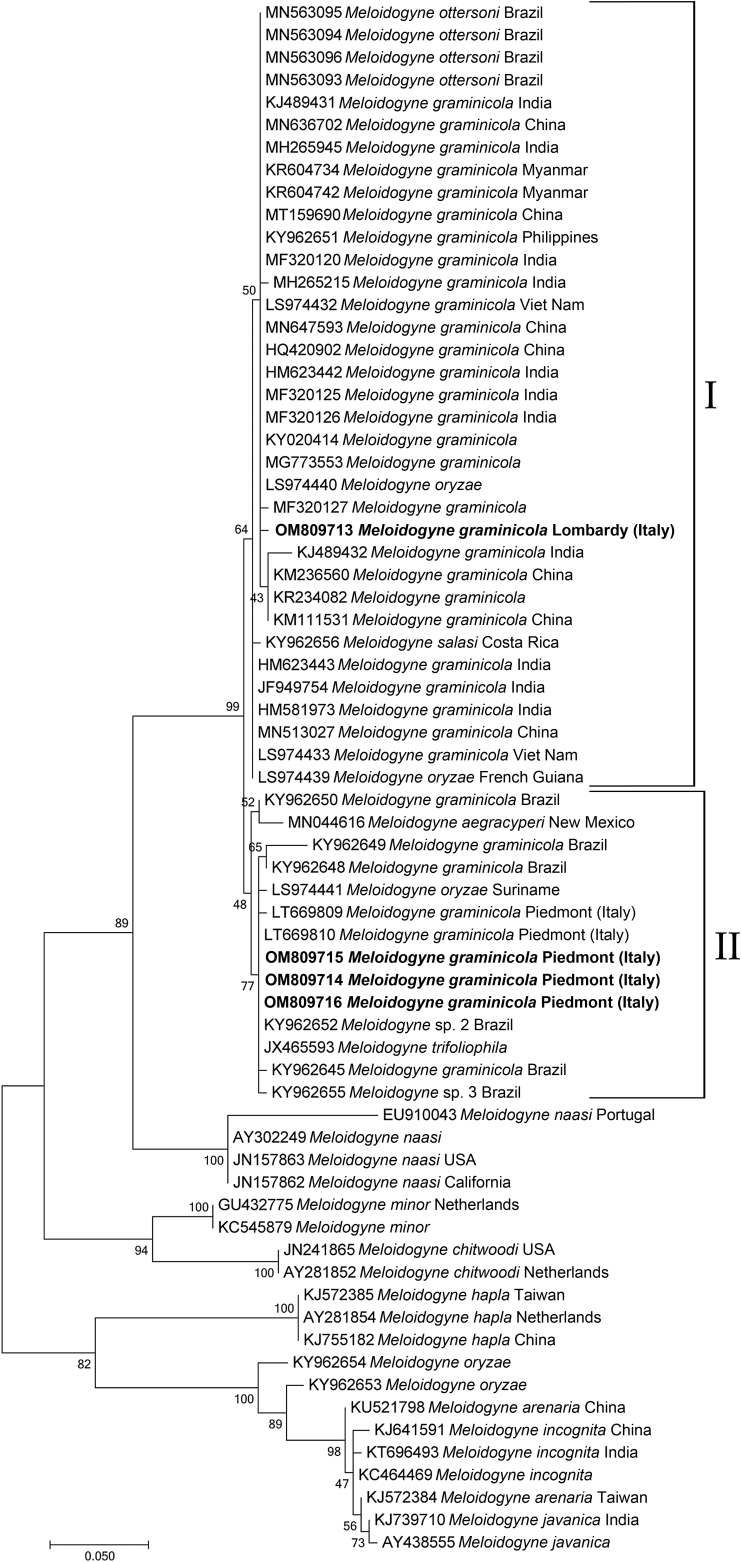
Phylogenetic tree of ITS containing region describing the evolutionary relationships among different geographical populations using Maximum Likelihood (ML) method. Branch lengths are proportional to the distances as derived from the distance matrix obtained using the GTR method with the invariant site plus gamma options. Numbers at nodes indicate bootstrap values. *Meloidogyne incognita*, *M. hapla*, *M. javanica*, and *M. arenaria* were used as outgroups. Newly obtained sequences are in bold. GenBank accession numbers are along with the species names.

Sixty‐nine sequences, including *M. graminicola* sequences from Lombardy and Piedmont (North Italy), are obtained in this study, and the corresponding sequences from Genbank, along with those of *Meloidogyne* spp. belonging to the *graminis* group and other *Meloidogyne* spp. from Genbank were aligned. Phylogenetic tree based on the ITS region revealed that *M. graminicola* from Italy clustered with all other *M. graminicola* and *Meloidogyne* spp. belonging to the *graminis* group (Figure 2). It is interesting to note that *M. graminicola* from Piedmont grouped in a subclade (83% support) with populations of *M. graminicola* from Brazil, *Meloidogyne* sp. 2, *Meloidogyne* sp. 3 from Brazil, *M. trifoliophila* and *M. oryzae* from Suriname, while *M. graminicola* from Lombardy with the rest of *M. graminicola* populations, *M. ottersoni* from Brazil, and *M. oryzae* from French Guiana. Closely related to *M. graminicola* group clustered *M. aegracyperi* (100% support), while *M. naasi*, belonging to the *graminis* group, clustered at a basal position of the *M. graminicola* and *M. aegracyperi* cluster. The two populations of *M. oryzae* from Brazil (KY962653‐KY962654; Mattos et al., 2018) grouped with mitotic parthenogenetic species (Figure 1; 98% support).

Two individual COI amplicons of *M. graminicola* from Lombardy were cloned and eight clones were sequenced. BLASTn analysis at NCBI revealed a 98–99% similarity with all *M. graminicola* sequences and 97–98% similarity with *M. oryzae*. Pairwise distances within the *graminis*‐group are shown in Table 1. Most of *M. graminicola* isolates differ by 0–7 bp each other, while with *M. graminicola* (MH128475; P169011 isolate) by 7–15 bp. All *M. oryzae* isolates differ by 5–16 bp from all *M. graminicola* isolates. COI sequences of *M. oryzae* (LR215847 and MK507908) were obtained from the complete and mitochondrial genomes, respectively (Besnard et al., 2014, 2019). Nucleotide sequences were also converted into amino‐acid sequences and no stop codons or frame shift mutations were observed. The amino‐acid analysis revealed that intraspecific amino‐acid sequence variation for *M. graminicola* isolates was only 1–2 amino acids, as that observed with *M. oryzae* isolates.

**TABLE 1:** Pairwise distances of mitochondrial COI of *Meloidogyne graminicola* populations in bold the sequences obtained in this study.

		1	2	3	4	5	6	7	8	9	10	11	12	13	14	15	16	17	18	19	20	21	22	23	24	25	26	27	28
1	**OM810293 *Meloidogyne graminicola* isolate Lombardy**	0																											
2	**OM810294 *Meloidogyne graminicola* isolate Lombardy**	0	0																										
3	**OM810295 *Meloidogyne graminicola* isolate Lombardy**	0	0	0																									
4	**OM810296 *Meloidogyne graminicola* isolate Lombardy**	0	0	0	0																								
5	**OM810297 *Meloidogyne graminicola* isolate Lombardy**	1	1	1	1	0																							
6	**OM810298 *Meloidogyne graminicola* isolate Lombardy**	1	1	1	1	2	0																						
7	**OM810299 *Meloidogyne graminicola* isolate Lombardy**	2	2	2	2	3	3	0																					
8	**OM810300 *Meloidogyne graminicola* isolate Lombardy**	0	0	0	0	1	0	1	0																				
9	LR215858 *Meloidogyne graminicola*	5	5	5	5	6	4	5	3	0																			
10	LR215853 *Meloidogyne graminicola*	5	5	5	5	6	4	5	3	0	0																		
11	LR215854 *Meloidogyne graminicola*	5	5	5	5	6	4	5	3	0	0	0																	
12	MH332672 *Meloidogyne graminicola*	4	4	4	4	5	5	6	4	3	3	3	0																
13	KY250093 *Meloidogyne graminicola*	4	4	4	4	5	5	6	4	3	3	3	0	0															
14	LR215857 *Meloidogyne graminicola*	6	6	6	6	7	5	6	3	0	0	0	3	3	0														
15	LR215848 *Meloidogyne graminicola*	6	6	6	6	7	5	6	3	0	0	0	3	3	0	0													
16	MW411965 *Meloidogyne graminicola*	6	6	6	6	7	5	6	3	0	0	0	3	3	0	0	0												
17	MN017128 *Meloidogyne graminicola*	5	5	5	5	6	4	7	3	2	2	2	3	3	2	2	2	0											
18	MG917045 *Meloidogyne graminicola*	3	3	3	3	4	3	4	3	0	0	0	1	1	0	0	0	0	0										
19	MG917043 *Meloidogyne graminicola*	0	0	0	0	1	0	1	0	0	0	0	1	1	0	0	0	0	0	0									
20	NC056772 *Meloidogyne graminicola*	6	6	6	6	7	5	6	3	0	0	0	3	3	0	0	0	2	0	0	0								
21	HG529223 *Meloidogyne graminicola*	7	7	7	7	8	6	7	4	1	1	1	4	4	1	1	1	3	1	1	1	0							
22	LR215855 *Meloidogyne graminicola*	3	3	3	3	4	2	2	0	0	0	0	3	3	0	0	0	2	0	0	0	1	0						
23	LR215851 *Meloidogyne graminicola*	6	6	6	6	7	5	6	3	0	0	0	3	3	0	0	0	2	0	0	0	1	0	0					
24	MH128473 *Meloidogyne oryzae*	11	11	11	11	12	10	11	8	7	7	7	10	10	7	7	7	9	7	5	7	8	5	7	0				
25	MH128474 *Meloidogyne oryzae*	11	11	11	11	12	10	11	8	7	7	7	10	10	7	7	7	9	7	5	7	8	5	7	0	0			
26	MH128475 *Meloidogyne graminicola*	13	13	13	13	14	14	15	11	14	14	14	13	13	14	14	14	12	11	7	14	15	9	14	15	15	0		
27	LR215847 *Meloidogyne oryzae* isolate Mo‐M1	14	14	14	14	15	13	15	12	10	10	9	12	12	10	10	10	11	9	7	10	11	7	10	12	12	14	0	
28	MK507908 *Meloidogyne oryzae* isolate Southeast Asia	15	15	15	15	16	14	16	12	10	10	9	12	12	10	10	10	11	9	7	10	11	7	10	12	12	14	0	0

**FIGURE 3:**
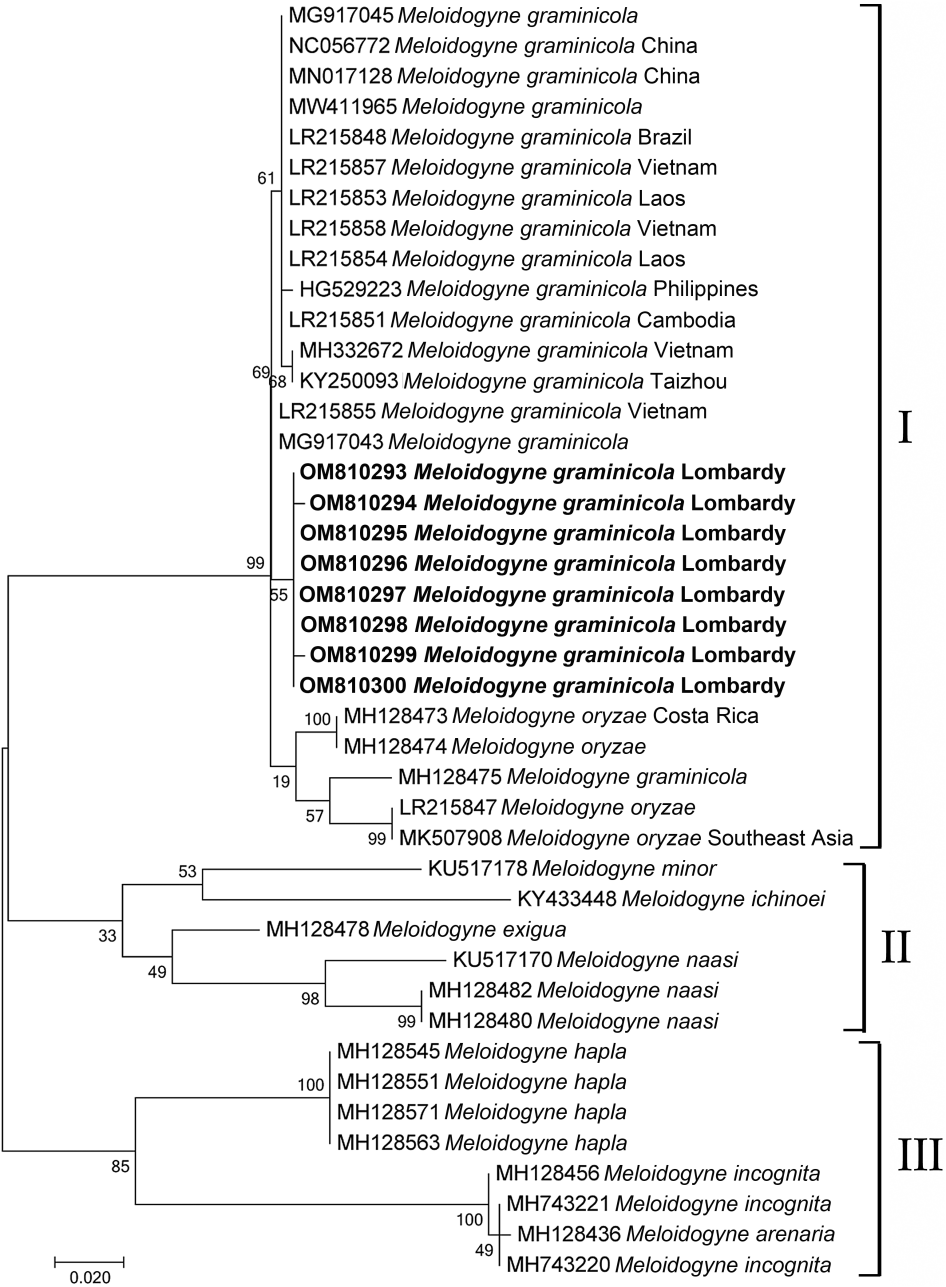
Phylogenetic tree of partial mitochondrial COI sequences describing the evolutionary relationships among different geographical populations of using Maximum Likelihood (ML) method. Branch lengths are proportional to the distances as derived from the distance matrix obtained using the GTR method with the invariant site plus gamma options. Numbers at nodes indicate bootstrap values. *Meloidogyne incognita*, *M. hapla*, *M. javanica*, and *M. arenaria* were used as outgroups. Newly obtained sequences are in bold. GenBank accession numbers are along with the species names.

Phylogenetic analysis based on mitochondrial COI sequences of *M. graminicola* from Lombardy and the corresponding available sequences of *graminis*‐group and other *Meloidogyne* spp. revealed three main groupings (Figure 3). The largest, Group I, contained all *M. graminicola* and *M. oryzae* haplotypes; Group II: *M*. *minor, M. ichinohei, M. exigua*, and *M. naasi* haplotypes; Group III: *M. hapla*, *M. incognita*, and *M. arenaria* haplotypes. Group I showed two subgroups: one containing all *M. graminicola* haplotypes including Lombardy haplotypes, the second subgroup containing *M. oryzae* isolates (M0‐M1, South East Asia, MH128473 and MH128474 isolates P129052, P129054, respectively), and *M. graminicola* (MH128475) isolate P169011 which could be misidentified.

The newly obtained COII sequences from Lombardy showed a low intraspecific variability, 1 to 2 bp out of 531 bp, while with the corresponding sequences of *M. graminicola* present in the database they showed 97–99% similarity (2 to 12 different nucleotides). Pairwise distances within the *graminis*‐group, using 396 sites, revealed that *M. graminicola* from Lombardy showed the highest variability, namely 8–10 different nucleotides with *M. graminicola* from Piedmont and *Meloidogyne* sp. 2 and sp. 3 (MN585713 and MN585714), while with *M. oryzae* (MN585712) from Brazil, French Guinea, and Southeast Asia (LR215847 and MK507948; mitochondrial complete genomes) 6–8 different nucleotides (Table 2).

**TABLE 2:** Pairwise distances of mitochondrial COII of *Meloidogyne graminicola* populations in bold the sequences obtained in this study.

		1	2	3	4	5	6	7	8	9	10	11	12	13	14	15	16	17	18	19	20	21	22	23	24	25
1	**OP024530 *Meloidogyne graminicola* isolate Lombardy**	0																								
2	**OP024532 *Meloidogyne graminicola* isolate Lombardy**	0	0																							
3	**OP024529 *Meloidogyne graminicola* isolate Lombardy**	1	1	0																						
4	**OP024528 *Meloidogyne graminicola* isolate Lombardy**	1	1	2	0																					
5	**OP024531 *Meloidogyne graminicola* isolate Lombardy**	0	0	1	1	0																				
6	**OP024535 *Meloidogyne graminicola* isolate Piedmont**	8	8	9	9	8	0																			
7	**OP024534 *Meloidogyne graminicola* isolate Piedmont**	8	8	9	9	8	0	0																		
8	LT669811 *Meloidogyne graminicola* isolate Piedmont	9	9	10	10	9	1	1	0																	
9	**OP024533 *Meloidogyne graminicola* isolate Piedmont**	8	8	9	9	8	0	0	1	0																
10	LT669812 *Meloidogyne graminicola* isolate Piedmont	8	8	9	9	8	0	0	1	0	0															
11	MH332687 *Meloidogyne graminicola* Vietnam	0	0	1	1	0	8	8	9	8	8	0														
12	KY020413 *Meloidogyne graminicola* China	0	0	1	1	0	8	8	9	8	8	0	0													
13	MG356944 *Meloidogyne graminicola* China	0	0	1	1	0	8	8	9	8	8	0	0	0												
14	JN241929 *Meloidogyne graminicola* USA	0	0	1	1	0	8	8	9	8	8	0	0	0	0											
15	JN241927 *Meloidogyne graminicola* Bangladesh	0	0	1	1	0	8	8	9	8	8	0	0	0	0	0										
16	LR215858 *Meloidogyne graminicola* Vietnam	0	0	1	1	0	8	8	9	8	8	0	0	0	0	0	0									
17	JN241939 *Meloidogyne graminicola* India	0	0	1	1	0	8	8	9	8	8	0	0	0	0	0	0	0								
18	KJ576894 *Meloidogyne graminicola* Taiwan	0	0	1	1	0	8	8	9	8	8	0	0	0	0	0	0	0	0							
19	MN585713 *Meloidogyne* sp. 2	8	8	9	9	8	4	4	5	4	4	8	8	8	8	8	8	8	8	0						
20	MN585715 *Meloidogyne* sp. 3	9	9	10	10	9	7	7	8	7	7	9	9	9	9	9	9	9	9	6	0					
21	MN585714 *Meloidogyne* sp. 3	9	9	10	10	9	7	7	8	7	7	9	9	9	9	9	9	9	9	6	0	0				
22	MN585720 *Meloidogyne salasi* Brazil	3	3	4	4	3	7	7	8	7	7	3	3	3	3	3	3	3	3	7	8	8	0			
23	MN585712 *Meloidogyne oryzae*	7	7	8	8	7	3	3	4	3	3	7	7	7	7	7	7	7	7	3	6	6	6	0		
24	LR215847 *Meloidogyne oryzae* isolate French Guiana	6	6	7	7	6	10	10	11	10	10	6	6	6	6	6	6	6	6	9	11	11	7	9	0	
25	MK507908 *Meloidogyne oryzae* isolate Southeast Asia	6	6	7	7	6	10	10	11	10	10	6	6	6	6	6	6	6	6	9	11	11	7	9	0	0

**FIGURE 4:**
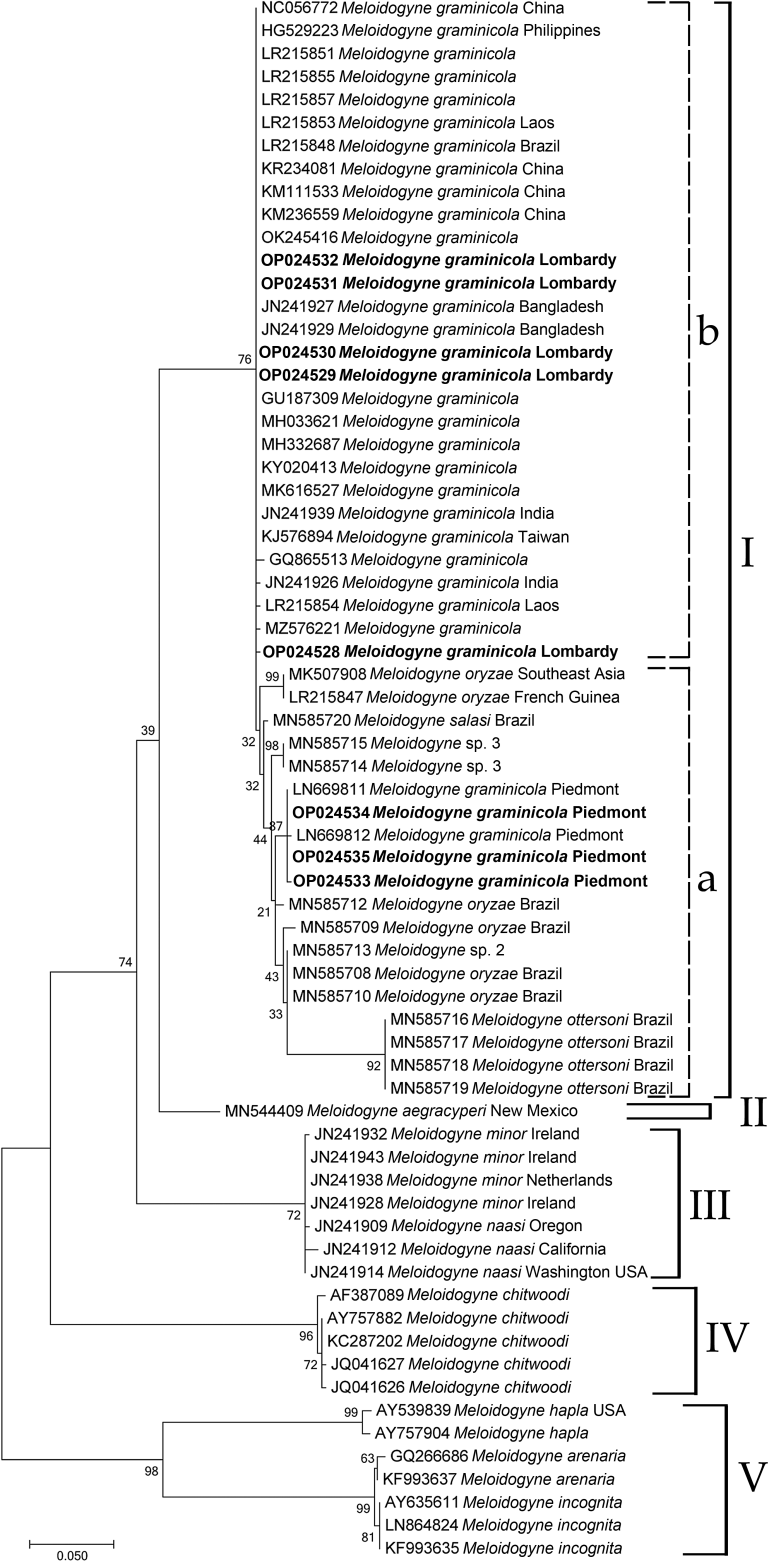
Phylogenetic tree of partial mitochondrial COII sequences describing the evolutionary relationships among different geographical populations of using Maximum Likelihood (ML) method. Branch lengths are proportional to the distances as derived from the distance matrix obtained using the GTR method with the invariant site plus gamma options. Numbers at nodes indicate bootstrap values. *Meloidogyne incognita*, *M. hapla*, *M. javanica*, and *M. arenaria* were used as outgroups. Newly obtained sequences are in bold. GenBank accession numbers are along with the species names.

For the COII‐16S‐rRNA, 68 sequences were aligned, 5 of which were from Lombardy population and 3 from upland rice field from Piedmont. Phylogenetic analysis is based on mitochondrial COII sequences of *M. graminicola* from Lombardy, and the corresponding available sequences of *graminis* group and other *Meloidogyne* spp. revealed five main groups (Figure 4). Group I showed two subgroups (99% support): subgroup A containing haplotypes of *M. graminicola* from Piedmont and Brazil, including *M. salasi*, *M. ottersoni*, *Meloidogyne* sp. 2 and sp. 3, haplotypes of *M. oryzae* from Brazil (MN585712), from French Guinea and Southeast Asia (MK507908 and LR215847, respectively); subgroup B containing all haplotypes of *M. graminicola* from Lombardy and Asian haplotypes (McClure et al., 2012). Group II contained *M. aegracyperi* at basal position of *Meloidogyne* spp. belonging to *graminis* group. Group III contained all haplotypes of *M. minor* and *M. naasi*. Group IV contained *M. chitwoodi*. Group V contained *M. hapla*, *M. arenaria*, and *M. incognita*.

## 4 DISCUSSION

In the present study, we report on the second occurrence of *M. graminicola* population in rice fields in Lombardy (North Italy), after the first occurrence in lowland and upland rice fields in Piedmont in 2016 (Fanelli et al., 2017). Piedmont and Lombardy regions are the main rice‐growing areas (more than 202,000 hectares, 93% of the Italian rice surface). In Piedmont region, most of the rice fields are under controlled field flooding, while in Lombardy region most of the rice fields are upland as the soil structure is characterized by a low water retention capacity (Sacchi et al., 2020) and water shortage as a result of local climate change (Zampieri et al., 2019). Recently, several species of *Meloidogyne* associated with rice causing severe damages were described (Kyndt et al., 2014; Mattos et al., 2018; Soares et al., 2020). In 2018, many rice fields in Lombardy showed the typical symptoms caused by *M. graminicola*: poor growth and stunting, clorosis, and heavily diseased roots with differently shaped and sized galls (Figure 1). The average measurements and the perineal patterns of females were in the range reported for *M. graminicola* in literature. Thus, the population of *M. graminicola* from Cascina Scalina field was characterized by sequencing the ITS, mitochondrial COI and COII to confirm the identification and assess its phylogenetic relationships with Piedmont and other *M. graminicola*. The ITS‐RFLP of Lombardy isolate produced restriction profiles identical to upland and lowland populations from Piedmont. Phylogenetic relationships based on ITS sequences using ML method confirmed that Lombardy population grouped with all *M. graminicola* sequences available in Genbank, but in a different subgroup compared with Piedmont *M. graminicola* isolates from upland and lowland rice fields (Figure 2). This finding confirms that *M. graminicola* populations do not group according to the geographical origin due to the high level of intraspecific variability. Thus, Lombardy population of *M. graminicola* could have been introduced independently from the Piedmont population confirming a recent expansion of this species in Italy and all over the world (Fanelli et al., 2017; Salalia et al., 2017; Soares et al., 2020). It is also noteworthy that in our phylogenetic analysis, *M. oryzae* isolates from French Guinea and Suriname, *M. trifoliophila* and *M. salasi* grouped within *M. graminicola* cluster confirming the occurrence of cryptic species and races of *M. graminicola* (Besnard et al., 2019; Mattos et al., 2018). The isolates of *M. oryzae* from Brazil clustered with mitotic parthenogenetic species showed 51–55 chromosomes, while *M. oryzae* isolates from French Guinea and Suriname showed 18 chromosomes (Mattos et al., 2018) corroborating the suspicious of Negretti et al. (2017) that these isolates (French Guinea and Suriname) could be misidentified (Carneiro et al., 2000) because the authors used only esterase phenotype diagnosis and thus they have to be considered as *M. graminicola* isolates (Alvarez‐Ortega et al., 2019; Mattos et al., 2018; Negretti et al., 2017). Recently, genome analyses of *M. graminicola* and *M. oryzae* reported by Besnard et al. (2019) revealed the presence of two divergent copies of ribosomal DNA in *M. graminicola* and two or three divergent copies in *M. oryzae*. One type of ribosomal sequence was shared by *M. graminicola* and *M. oryzae* suggesting that it could arise by hybridization or duplication (Alvarez‐Ortega et al., 2019; Besnard et al., 2019; Hugall et al., 1994; Phan et al., 2020; Szitenberg et al., 2017). These findings overall suggest a closely related ancestor of *M. graminicola* and *M. oryzae*. In the present study, the mitochondrial COI and COII‐16SrRNA genes were also used to investigate the genetic diversity among Italian and other isolates of *M. graminicola* and the geographical origin of Italian populations. Mitochondrial DNA shows high mutation rate and thus is a powerful marker to detect the intraspecific and interspecific variability in order to distinguish closely related and cryptic species (Blok & Powers, 2009; Powers et al., 2018). COI barcode, as a protein coding gene, is a powerful region to identify and to recognize groupings at species level. The phylogenetic tree based on COI sequences revealed that all haplotypes from Lombardy population formed a supported subgroup within *M. graminicola* group, whereas all *M. oryzae* isolates were positioned at basal position of the *M. graminicola* group (Figure 3). To further evaluate the genetic diversity and phylogenetic relationships between *M. graminicola* and *M. oryzae*, the ML COXII‐16SrRNA genes tree revealed that Lombardy haplotypes grouped in a subgroup with Asian *M. graminicola* haplotypes sharing common COII haplotypes with geographical distant populations, while Piedmont haplotypes formed a separated subgroup with *M. graminicola* haplotypes from Brazil, *M. oryzae*, *M. salasi*, *M. ottersoni*, *Meloidogyne* sp. 2 and *Meloidogyne* sp. 3 (Figure 4). Furthermore, COII Piedmont haplotypes belonged to type A for the presence of one *Dra* I and four *Ssp* I restriction sites, while those from Lombardy belonged to type B with two *Dra* I and four *Ssp* I restriction sites.

These findings suggest that geographical distance is not the main factor leading to *M. graminicola* population differentiation. As the Italian isolates always grouped in two different subgroups with geographical distant isolates sharing the same haplotypes, this observation seems to confirm that the two Italian *M. graminicola* populations may have been recently and independently introduced. Furthermore, in the current study, some *M. oryzae* isolates grouped within *M. graminicola* cluster confirming their close relationships due to recent evolution or hybrid origin of these specie well adapted to irrigated rice (Besnard et al., 2019).

In conclusion, the present study clearly demonstrates that Italian *M. graminicola* populations show the same genetic profiles of those from Asia and America suggesting that this species prefers asexual reproduction and is well adapted to different rice fields. In this context, it can be understood that appropriate control measures are needed to manage this pest. Rice field flooding seems to be an efficient control technique for *M. graminicola* in Piedmont but not in Lombardy due to the soil structure. Thus, in Lombardy, researchers are testing several trap cropping strategies to maintain a low nematode population in infested fields and also balancing water shortage due to climate change and dry‐seeding practices. Regarding the entry of *M. graminicola* in Italy, it is not well understood yet. It seems through the movement of infested host plants, soil, waterbirds, acquatic plants or weeds acting as reservoirs for this nematode.

## AUTHOR CONTRIBUTIONS

**Elena Fanelli:** Conceptualization (equal); formal analysis (lead); investigation (equal); validation (equal); writing – original draft (equal). **Francesca Gaffuri:** Formal analysis (equal); methodology (equal); writing – original draft (equal). **Alberto Troccoli:** Formal analysis (supporting); writing – original draft (supporting). **Stefano Sacchi:** Methodology (equal); writing – original draft (supporting). **Francesca De Luca:** Conceptualization (lead); formal analysis (equal); funding acquisition (lead); project administration (lead); supervision (lead); writing – original draft (lead); writing – review and editing (lead).

## CONFLICT OF INTEREST

The authors declare no conflict of interest.

## Data Availability

DNA sequences obtained in the present study are available at Genbank accession numbers: for ITS OM809713‐OM809716; for COI OM810293‐OM810300; for COII OP024528‐OP024535.
